# Gut microbial DNA and immune checkpoint gene Vsig4/CRIg are key antagonistic players in healthy aging and age-associated development of hypertension and diabetes

**DOI:** 10.3389/fendo.2022.1037465

**Published:** 2022-11-11

**Authors:** Matthew A. Liu, Shandy Shahabi, Suborno Jati, Kechun Tang, Hong Gao, Zhongmou Jin, Wyatt Miller, Frédéric A. Meunier, Wei Ying, Geert van den Bogaart, Gourisankar Ghosh, Sushil K. Mahata

**Affiliations:** ^1^ Department of Medicine, University of California, San Diego, La Jolla, CA, United States; ^2^ Department of Chemistry and Biochemistry, University of California, San Diego, La Jolla, CA, United States; ^3^ Veterans Affairs (VA) San Diego Healthcare System, San Diego, CA, United States; ^4^ Clem Jones Center for Ageing Dementia Research, Queensland Brain Institute, The University of Queensland, Brisbane, QLD, Australia; ^5^ Department of Molecular Immunology and Microbiology, Groningen Biomolecular Sciences and Biotechnology Institute, University of Groningen, Groningen, Netherlands

**Keywords:** Chromogranin A, catestatin, hypertension, pancreastatin, insulin resistance, diabetes, healthy aging

## Abstract

**Aims:**

Aging is associated with the development of insulin resistance and hypertension which may stem from inflammation induced by accumulation of toxic bacterial DNA crossing the gut barrier. The aim of this study was to identify factors counter-regulating these processes. Taking advantage of the Chromogranin A (CgA) knockout (CgA-KO) mouse as a model for healthy aging, we have identified *Vsig4* (V-set and immunoglobulin domain containing 4) as the critical checkpoint gene in offsetting age-associated hypertension and diabetes.

**Methods and Results:**

The CgA-KO mice display two opposite aging phenotypes: hypertension but heightened insulin sensitivity at young age, whereas the blood pressure normalizes at older age and insulin sensitivity further improves. In comparison, aging WT mice gradually lost glucose tolerance and insulin sensitivity and developed hypertension. The gut barrier, compromised in aging WT mice, was preserved in CgA KO mice leading to major 35-fold protection against bacterial DNA-induced inflammation. Similarly, RNA sequencing showed increased expression of the *Vsig4* gene (which removes bacterial DNA) in the liver of 2-yr-old CgA-KO mice, which may account for the very low accumulation of microbial DNA in the heart. The reversal of hypertension in aging CgA-KO mice likely stems from (i) low accumulation of microbial DNA, (ii) decreased spillover of norepinephrine in the heart and kidneys, and (iii) reduced inflammation.

**Conclusion:**

We conclude that healthy aging relies on protection from bacterial DNA and the consequent low inflammation afforded by CgA-KO. *Vsig4* also plays a crucial role in “healthy aging” by counteracting age-associated insulin resistance and hypertension.

## Introduction

The past 150 years have witnessed a dramatic increase in life expectancy (1). However, the proportion of life in good health has remained broadly constant, implying increasing years in poor health (2). Therefore, there is a growing emphasis to look for factors that promote “healthy aging” (3–5).

Two common diseases are hypertension and diabetes. Both are more prevalent in the older population. The prevalence of hypertension increases with age, affecting two-thirds of people aged >60 years (6, 7). Type 2 diabetes (T2D), the most prevalent form of diabetes in older adults, is also an age-related disorder, which stems from the combined effects of genetics, lifestyle, and age. The following mechanisms have been implicated in age-related development of T2D: (i) defects in insulin signaling (8), (ii) a decrease in insulin-stimulated whole-body glucose oxidation (8), (iii) a reduction in the β-cell response to glucose (8), (iv) impaired insulin-mediated glucose uptake (9), and (v) an inability to suppress hepatic glucose production (9). Despite extensive investigation, the factors responsible for this aging have remained elusive.

The current study examined the pro-hormone Chromogranin A (CgA), as this protein regulates both hypertension and diabetes. As a pro-hormone, CgA gives rise to several counterregulatory peptides upon proteolytic digestion, such as an anti-diabetic and anti-hypertensive peptide catestatin (CST) (10), as well as pro-diabetic and pro-hypertensive peptide pancreastatin (PST) (11). We hypothesized that CST and PST affect healthy aging, due to their metabolic and immune-modulatory effects. For instance, the lack of PST makes CgA-KO mice sensitive to insulin (12, 13) and supplementation of CgA-KO mice with PST makes them resistant to insulin (14, 15), implicating PST as a pro-diabetic peptide. Conversely, lack of CST in the presence of PST makes CST-KO resistant to insulin and supplementation of CST-KO mice with CST improved insulin sensitivity in CST-KO mice (16). Moreover, CST also improves insulin sensitivity in diet-induced obese and insulin-resistant mice (16). These findings point to CST as an anti-diabetic peptide. Furthermore, supplementation of CgA-KO and CST-KO mice with CST normalizes blood pressure (12, 13) and treatment of blood pressure high mice (17) and spontaneously hypertensive rats (18) with CST reduces blood pressure, associating CST as an anti-hypertensive peptide. CST also acts as a cardioprotective peptide (19–21). In addition to diabetes and hypertension, one of the major changes that occurs during aging is the dysregulation of the immune response, leading to a chronic systemic inflammatory state (22–24). CST and PST also regulate inflammation, and for example CST-KO mice have elevated expression of inflammatory genes and macrophage infiltration in the heart, which can be reversed by administration of CST (16).

The present study is based on our observation that the immune checkpoint gene *Vsig4* (V-set and immunoglobulin domain containing 4), is expressed at much higher levels in the liver of CgA-KO mice. *Vsig4*, expressed mainly in liver resident Kupffer cells, encodes a protein called CRIg (complement receptor immunoglobulin) (25, 26), which removes gut bacterial DNA and bacterial byproducts through C3 (complement 3)-mediated opsonization (27–29). Therefore, low levels of expression of Vsig4/CRIg can be expected to result in accumulation of bacterial DNA and byproducts in the liver and other distant tissues and cause tissue inflammation over time. We therefore hypothesized that aging WT mice would show systemic inflammation, whereas this would be less in aging CgA-KO mice. The present study identified Vsig4/CRIg as a key player in “healthy aging”.

## Research design and methods

### Animals and diets

Male WT and CgA-KO (0.5 to 2 yrs old) were in C57BL/6 background. Since CgA is especially overexpressed in male patients with hypertension (30), we used only male mice in this study. Mice were kept in a 12 hr dark/light cycle and fed a normal chow diet (NCD: 13.5% calorie from fat; LabDiet 5001, TX). Animals were age-matched, and randomly assigned for each experiment. Control and experimental groups were blinded. All studies with mice were approved by the UCSD and Veteran Affairs San Diego Institutional Animal Care and Use Committees and conform to relevant National Institutes of Health guidelines.

### Quantification of bacterial DNA

Bacterial DNA was extracted from heart samples using the ZymoBIOMICS DNA extraction kit (catalog #D4301; Zymo Research) according to the manufacturer’s instructions. Levels of bacterial DNA were determined by qPCR using a Femto Bacterial DNA Quantification kit (catalog #E2006; Zymo Research) by following the manufacturer’s instructions.

### RNA isolation and sequencing

Total RNA was isolated from approximately 30 mg of liver tissue from 3 wild type and 3 CgA knockout mice using RNeasy Mini Kit (Qiagen) following the manufacturer’s instructions. Total RNA was quantified by nanodrop and was quality checked by Tapestation (Agilent). Poly(A) selected mRNA libraries were prepared from 500 ng of total RNA using KAPA mRNA HyperPrep kit (KAPA Biosystems) and dsDNA libraries were quantified using Qubit 2.0 fluorometer (Invitrogen). Libraries were pooled and sequencing was performed on Illumina NovaSeq S2 with paired end 100bp sequencing, according to manufacturer’s recommendations by the Institute for Genomic Medicine (IGM) Genomics Center at the University of California, San Diego.

### RNA-seq data analysis

Raw read counts were quantified at the transcript level and aligned to the GRCm38 mouse transcriptome using Salmon v1.4.0. Read counts less than 1 were excluded in downstream analysis. The Bioconductor-DESeq2 package v1.36.0 was used for differential gene expression analysis using R v4.2.0. A heatmap was generated using the pheatmap package. Volcano plot was prepared with the EnhancedVolcano package with a combination of log_2_ fold change value > 1.5 and log_10_ p-value < -7 as a threshold for significance. Gene set enrichment analysis was performed using the fgsea package with 1000 permutations and an adjusted p-value threshold < 0.05 with the “Hallmark” gene set collection from the Molecular Signatures Database v7.5.1.

### Real time PCR

Total RNA from liver tissue was isolated using RNeasy Mini Kit and reverse-transcribed using a qScript cDNA synthesis kit. cDNA samples were amplified using PERFECTA SYBR FASTMIX L-ROX 1250 and analyzed on an Applied Biosystems 7500 Fast Real-Time PCR system. All PCRs were normalized to *Rplp0* (Ribosomal protein, large, P0), and relative expression levels were determined by the ΔΔ*C_t_* method.

### Tail-cuff measurement of blood pressure

Systolic blood pressure (SBP) was measured using the mouse and rat tail cuff blood pressure (MRBP) System (IITC Life Sciences Inc. Woodland Hills, CA). Mice were restrained in plexiglass tubes and heated to 34°C for 10-15 min in individual warming chambers prior to BP measurement. The tails were placed inside inflatable cuffs with a photoelectric sensor that measured tail pulses. The SBP was measured over 6 separate days with an average of two values per day.

### Glucose tolerance test, glucose-stimulated insulin secretion, and insulin tolerance test

For GTT, glucose (1 mg/g body weight) was injected intraperitoneally (time zero) after an 8-hr fast. Tail-vein glucose levels were measured at 0, 15, 30, 60, 90, and 120 min. For GSIS, blood was collected from the tail-vein at 0 and 10 min and the plasma was used for an insulin assay. For insulin tolerance tests (ITT), insulin (0.4 mU/g body weight) was injected intraperitoneally, and blood glucose levels were measured at the same time points as for GTT.

### Protein analysis by immunoblotting

Liver pieces were homogenized in a buffer (50 mM Tris–Cl pH 8.0, 150 mM NaCl, 1% Triton X-100, 0.5% sodium deoxycholate, 0.1% SDS, 50 mM DTT, 5% glycerol, 5 mM NaF, 2 mM Na_3_VO_4_, 50 mM PMSF, 1 mM EDTA, Protease Inhibitor Cocktail from Sigma Aldrich) as previously described (17). Liver homogenates were subjected to SDS-PAGE and immunoblotted with antibodies directed against Phospho-Ser473-AKT (1:2000; catalog #9271S) and total AKT (1:4000; catalog #4685S), phospho-Ser9-GSK-3β (1:2000; catalog #9322S) and total GSK-3β (1:4000; catalog #9832S), phospho-Thr180/Tyr182-p38 (1:2000; catalog #9211S) and total p38 (1:5000; catalog #9212S) as well as phospho-Thr202/Tyr204-ERK1/2 (1:1000; catalog #4377S) and total ERK1/2 (1:3000; catalog #4695S). All these primary antibodies were purchased from Cell Signaling Technology (Danvers, MA). Anti-rabbit IgG-HRP conjugate (A6154; 1:8000) and anti-mouse IgG-HRP conjugate (A9044; 1:8000) were purchased from Sigma-Aldrich (St. Louis, MO).

### Electron microscopy

To displace blood and wash off tissues before fixation, mice were cannulated through the apex of the heart and perfused with a calcium and magnesium free buffer composed of DPBS (Life Technologies Inc), 10 mM HEPES, 0. 2 mM EGTA, 0.2% BSA, 5 mM glucose and KCl concentration adjusted 9.46 mM (to arrest the heart in diastole) as described previously (31). This was followed by perfusion fixation with freshly prepared fixative containing 2.5% glutaraldehyde, 2% paraformaldehyde in 0.15 M cacodylate buffer, and post fixed in 1% OsO_4_ in 0.1 M cacodylate buffer for 1 hour on ice. After perfusion, small pieces of the liver were immersed in the above fixative for 12-16 hrs. The tissues were stained *en bloc* with 2-3% uranyl acetate for 1 hour on ice. The tissues were dehydrated in graded series of ethanol (20-100%) on ice followed by one wash with 100% ethanol and two washes with acetone (15 min each) and embedding with Durcupan. Sections were cut at 50 to 60 nm on a Leica UCT ultramicrotome and picked up on Formvar and carbon-coated copper grids. Sections were stained with 2% uranyl acetate for 5 minutes and Sato’s lead stain for 1 minute. Grids were viewed using a JEOL JEM1400-plus TEM (JEOL, Peabody, MA) and photographed using a Gatan OneView digital camera with 4k x 4k resolution (Gatan, Pleasanton, CA).

### Measurement of cytokines

Plasma (20 µl) cytokines were measured using U-PLEX mouse cytokine assay kit (Meso Scale Diagnostics, Rockville, MD) *via* the manufacturer’s protocol.

### Statistics

Statistics were performed with PRISM 8 (version 8.4.3) software (San Diego, CA). Data were analyzed using unpaired two-tailed Student’s t-test for comparison of two groups or 1-way/2-way/3-way analysis of variance (ANOVA) for more than two groups followed by Tukey’s *post hoc* test if appropriate. All data are presented as mean ± SEM. Significance was assumed when p<0.05.

## Results

### Enrichment of bacterial DNA in 2 yr old WT heart resulting from leaky gut

**Figure 1:**
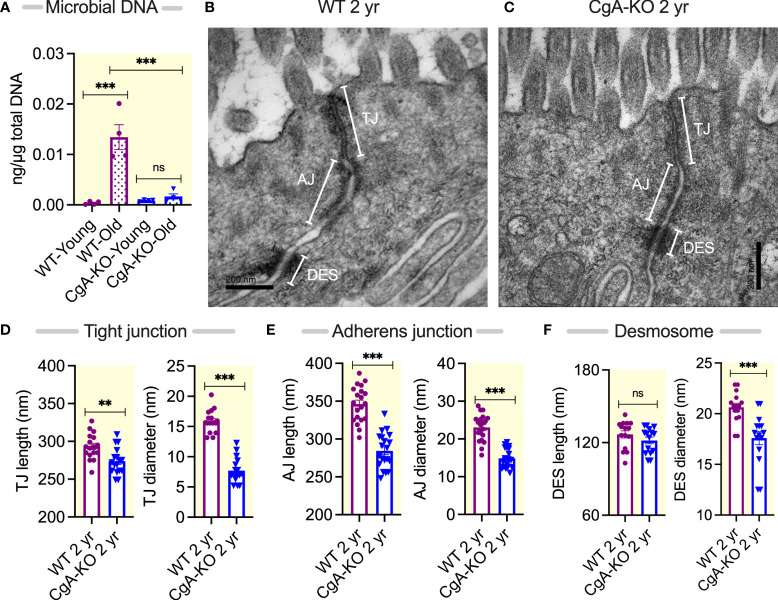
Age-associated changes in tissue bacterial DNA and gut barrier components. **(A)** Bacterial DNA in the heart of young (0.5 yr) and old (2 yr) WT and CgA-KO mice. **(B, C)** Electron micrographs showing gut barrier in colon of 2 yr old WT and CgA-KO mice. **(D–F)** Morphometric analyses of the gut barrier showing the lengths and diameters of tight junctions **(D)**, adherens junction **(E)**, and desmosomes **(F)**. AJ, adherens junction; Des, desmosomes; MV, microvilli; TJ, tight junction. **P <0.01; ***P <0.001.

Since obesity has been shown to be associated with the accumulation of bacterial DNA in host circulation and tissues (29, 32, 33), we assessed whether bacterial DNA is accumulated in the heart upon aging. Compared to young (0.5 year) WT mice and to both young and old (2 year) CgA-KO mice, quantitative RT-PCR analysis showed an increased (~35-fold) abundance of bacterial DNA in the heart of 2 yr old WT mice (**Figure 1A**).

Since a “leaky gut” facilitates the accumulation of bacterial DNA in the host circulation and tissues (34–36), we evaluated the gut barrier at the ultrastructural level. We found an increased length and diameter of tight junctions and adherens junctions, and an increased diameter in desmosomes in 2 yr old WT mice compared to 2 yr old CgA-KO mice, indicating a “leaky gut” in WT mice (**Figures 1B–F**).

### Bacterial DNA-induced inflammation and hypertension in aging WT mice

**Figure 2:**
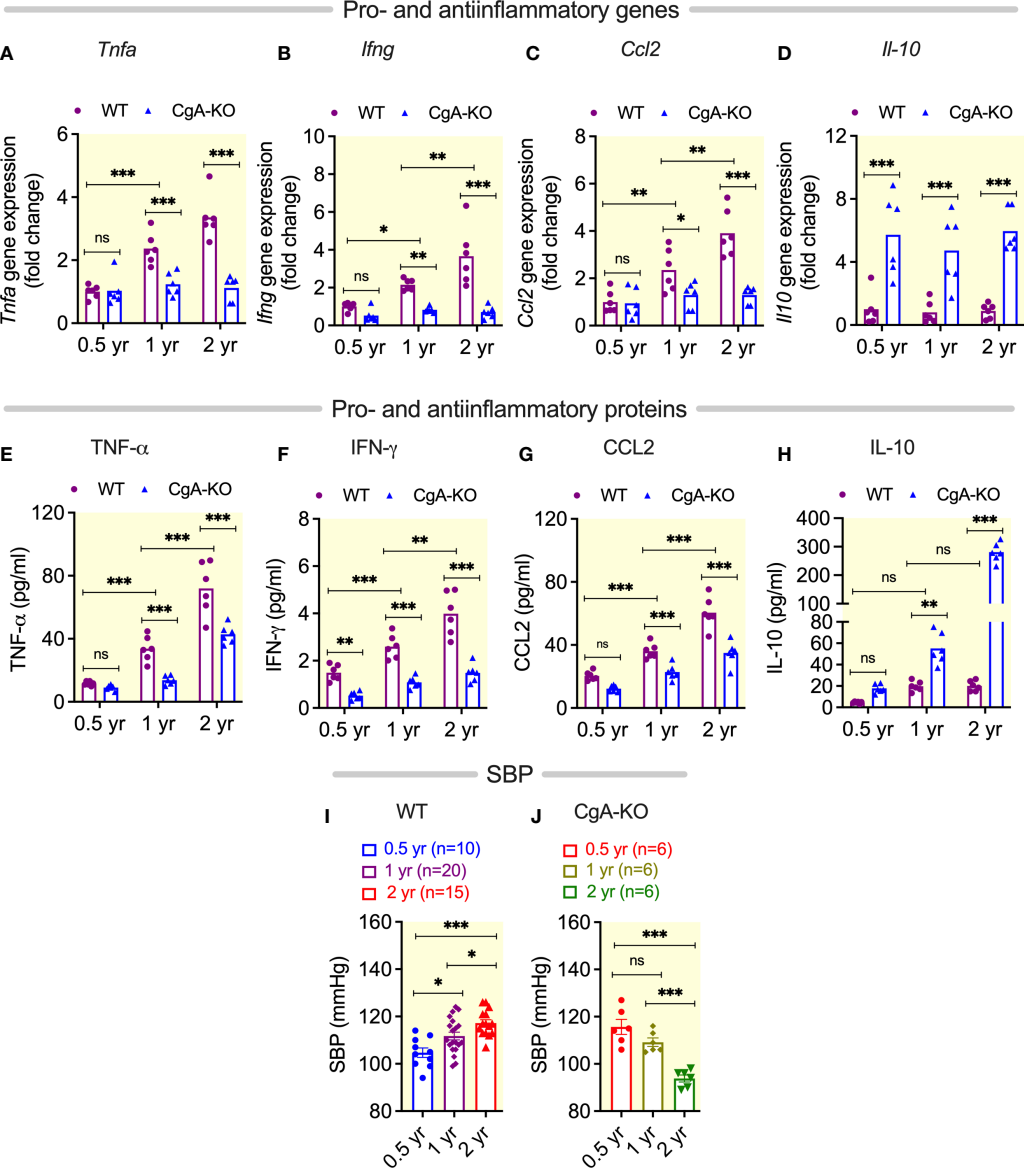
Age-associated Inflammation and blood pressure. **(A–D)** Steady-state mRNA levels of hepatic pro-inflammatory genes **(A)** *Tnfa*, **(B)** *Ifng*, and **(C)** *Ccl2*, as well as anti-inflammatory gene **(D)** *Il10*. **(E–H)** Plasma levels of pro-inflammatory proteins **(E)** TNF-α, **(F)** IFN-γ, and **(G)** CCL2, as well as anti-inflammatory protein **(H)** IL10. Blood pressure in **(I)** WT and **(J)** CgA-KO mice. *P <0.05; **P <0.01; ***P <0.001.

We have recently reported that enrichment of microbial DNA in the adrenal medulla of obese mice causes inflammation, increased catecholamine secretion and the consequent development of hypertension (37). Since we observed an increased accumulation of bacterial DNA in the heart of 2 yr old WT mice, we examined the levels of pro-inflammatory cytokines in the liver and plasma. We found increased expression of the proinflammatory genes *Tnfa* (tumor necrosis factor alpha), *Ifng* (interferon gamma) and *Ccl2* (chemokine C-C motif ligand 2) in the liver of 1 and 2 yr old WT mice (**Figures 2A–C)**. The levels in the plasma of the corresponding proteins TNFα, IFNγ, and CCL2 were also elevated (**Figures 2E–G**). Expression of anti-inflammatory IL-10 was not significantly altered in aging WT mice (**Figures 2D, H**). Consistent with inflammation-induced hypertension (38, 39), we found an increased blood pressure in aging WT mice (**Figure 2I**).

### Decreased inflammation and spontaneous reversal of hypertension in aging CgA-KO mice

Since aging CgA-KO mice showed only a ~2-fold accumulation of bacterial DNA compared to a ~35-fold in WT heart, we expected a decreased inflammation in CgA-KO mice compared to WT mice. Consistent with this hypothesis, we found a lower hepatic expression of proinflammatory genes *Tnfa*, *Ifng* and *Ccl2* (**Figures 2A–C**) and a higher expression of anti-inflammatory gene *Il10* in CgA-KO mice (**Figure 2D**). Similar results were detected in plasma protein levels (**Figures 2E–H**). These results show that whereas WT mice displayed increased inflammation upon aging, this was not present in CgA-KO mice. Strikingly, the high blood pressure in young CgA-KO mice (12) was spontaneously reversed in 2-yr-old CgA-KO mice (**Figure 2J**).

### RNA-seq analyses of liver genes identified genes responsible for age-associated hypertension and diabetes

**Figure 3:**
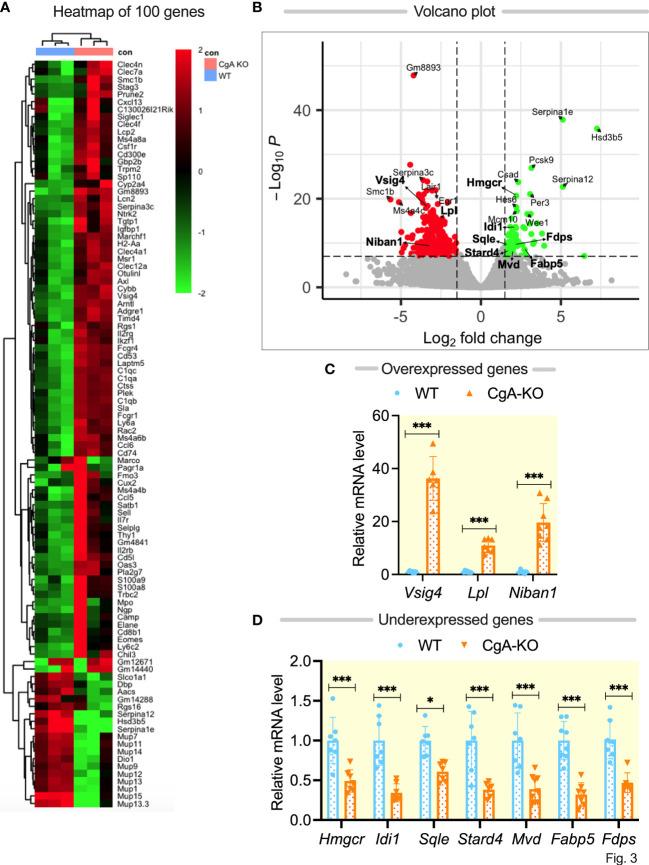
RNA sequencing of liver genes followed by q-RT-PCR validation of selected genes. **(A)** Heat map showing differentially expressed top 100 genes in WT and CgA-KO mice. **(B)** Volcano plot showing expression (over- and under-expression) of selected genes. **(C)** q-RT-PCR validation of selective overexpressed genes in CgA-KO mice as compared to WT mice. **(D)** q-RT-PCR validation of seven under-expressed genes in CgA-KO mice compared to WT mice. *P <0.05; ***p <0.001.

To find out the mechanisms underlying spontaneous reversal of hypertension in CgA-KO mice, we focused on global transcriptome changes in the livers of 0.5-, 1- and 2-year-old WT and CgA-KO mice by RNA-seq. Differential expression analysis of pairwise comparison of CgA-KO versus WT identified 2276 differentially expressed genes (DEGs) with a false discovery rate (FDR/padj) <0.05. The heat maps of the top 100 DEGs in individual samples in each WT and KO groups are shown in **Figure 3A**. The clear separation of the genotype expression levels is evident in the heatmap. To investigate the functional associations of the common DEGs, we performed GO analysis using Gene Ontology. GO analysis based on biological pathways revealed 11 enriched pathways from up-regulated DEGs and 23 enriched pathways from down-regulated DEGs. The expression (over- and under-expression) of selected genes was shown in a Volcano plot (**Figure 3B**). Metabolic, and inflammatory pathways were significantly different in these two groups.

We chose to further validate the DEG by qPCR targeting genes in the cholesterol metabolism: 3-hydroxy-3-methylglutaryl-Coenzyme A reductase (*Hmgcr*), lipoprotein lipase (*Lpl*), isopentenyl-diphosphate delta isomerase (*Idi1*), farnesyl diphosphate synthetase (*Fdps*), Squalene epoxidase (*Sqle*), StAR-related lipid transfer (START) domain containing 4 (*Stard4*), Niban apoptosis regulator 1 (*Niban 1*), fatty acid binding protein 5 (*Fabp5*), and mevalonate (diphospho) decarboxylase (*Mvd*), since the p and padj values of these selected genes are <10^-7^ (**Figures 3C, D**). Importantly, these genes have been shown to be associated with insulin’s mechanism of action. Pathways with upregulated DEGs are involved in insulin resistance whereas downregulated inflammatory pathways as revealed by KO are responsible for insulin sensitivity. For instance, high *Hmgcr* induces insulin resistance (40, 41), whereas high *Lpl* protects against insulin resistance in high-fat diet fed mice (42, 43). *Niban1* promotes survival during stress (44). *Both* Lpl and *Niban1* are highly expressed in CgA KO mice, whereas *Hmgcr* is expressed at lower levels. Another gene which is highly overexpressed in KO liver is *Vsig4*. Consistent with the genome-wide data, qPCR showed that the expression of these genes is different between WT and KO livers. The highest expression (by ~36-fold) was seen for the *Vsig4* gene in 2-yr-old CgA-KO compared to WT mice (**Figure 3C**).

### Heightened sympathetic stimulation in 2 yr old WT heart and kidney

**Figure 4:**
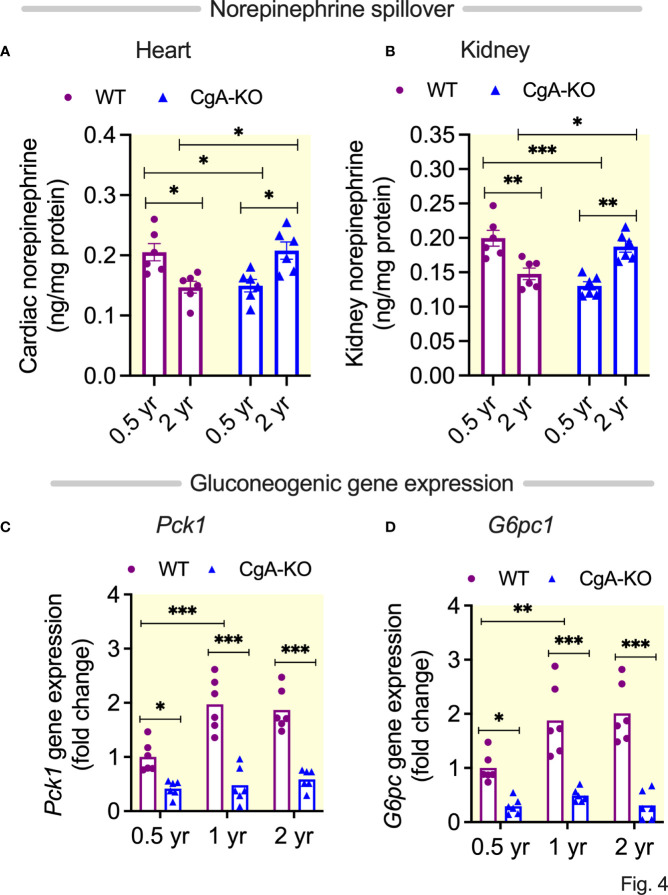
Spillover of norepinephrine and expression of gluconeogenic genes. Spillover of norepinephrine in **(A)** Heart, and **(B)** Kidney of 0.5, 1 and 2 yr old WT and CgA-KO mice. Expression of hepatic gluconeogenic genes: **(C)** *Pck1*, and **(D)** *G6pc*. *P <0.05; **P <0.01; ***P <0.001.

Existing literature describes heightened sympathetic nerve traffic in hypertensive patients (45). Therefore, we asked whether decreased spillover of norepinephrine (NE) in heart and kidney could explain spontaneous reversal of hypertension in aging CgA-KO mice. We found decreased NE concentration in the heart and kidney of 2 yr old WT mice and young CgA-KO mice (**Figures 4A, B**). Decreased NE in old WT and young CgA-KO mice indicates increased cardiac and renal spillover of NE, which is common in hypertensive and heart failure patients (46–48). In contrast, the spillover of NE was markedly reduced in 2 yr old CgA-KO mice and was comparable to 0.5 yr old normotensive WT mice (**Figures 4A, B**).

### Suppression of hepatic glucose production in aging CgA-KO mice

One of the main functions of insulin is to suppress hepatic glucose production (HGP), which is known to be compromised in the elderly population (49). Consistent with literature (49), our data show increased expression of gluconeogenic genes *Pck1* (phosphoenolpyruvate carboxykinase 1) and *G6pc* (glucose-6-phosphatse) in the liver of aging (1 and 2 yr old) WT mice (**Figures 4C, D**). In contrast, this increase was not observed in aging CgA-KO mice (**Figures 4C, D**), in line with previous results (14).

### Aging CgA-KO mice display improved insulin sensitivity

**Figure 5:**
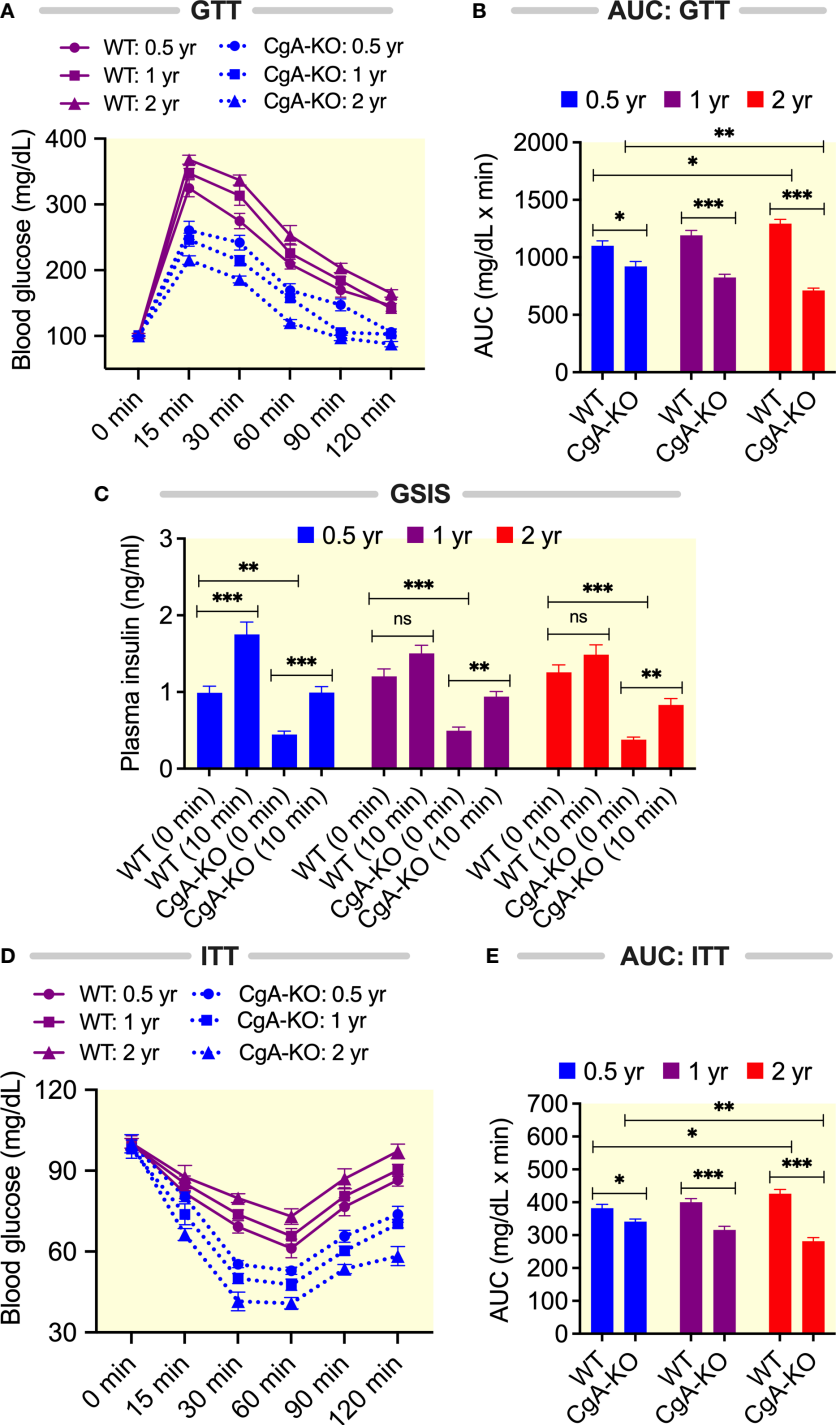
Aging CgA-KO mice maintain and improve glucose tolerance and insulin sensitivity. **(A)** Glucose tolerance test (GTT, n=7) with 8h fasting 0.5, 1 and 2 yr old WT and CgA-KO mice and **(B)** the corresponding areas under the curve (AUC). 2-way ANOVA. Interaction: p<0.001; Time: p<0.001; Genotype and age: p<0.001. **(C)** Glucose-stimulated insulin secretion (GSIS) with 8h fasting mice. Data were analyzed by 3-way ANOVA: Age: ns; Genotype; p<0.001; Treatment: p<0.001; Age: x Genotype: ns; Age x treatment: p<0.05; Genotype: x Treatment: ns; Age x Genotype x Treatment: ns. **(D)** Insulin tolerance test (ITT, n=7) on 8h fasting mice and **(E)** the corresponding AUC. 2-way ANOVA. Interaction: p<0.001; Time: p<0.001; Genotype and age: p<0.001. *P < 0.05; **P < 0.01; ***P < 0.001.

It is well documented that aging is associated with declines in glucose tolerance and insulin sensitivity in both mice (50) and humans (51). We have reported previously that young (~4-6 months old) CgA-KO mice on both a normal chow diet (NCD) and a high fat diet display heightened insulin sensitivity (14, 15). Therefore, we asked the question whether CgA-KO mice could maintain improved insulin sensitivity with aging. As expected (14), the glucose tolerance test (GTT) revealed a gradual decrease in glucose tolerance with aging in WT mice (**Figures 5A, B**). Likewise, glucose-stimulated insulin secretion (GSIS) was reduced in 1 and 2 yr old WT mice (**Figure 5C**). In contrast, glucose tolerance improved and GSIS was maintained in aging CgA-KO mice (**Figures 5A–C**). The insulin tolerance test (ITT) showed a progressive deterioration of insulin sensitivity in aging (0.5 yr to 2 yr) WT mice (**Figures 5D, E**). In contrast, aging CgA-KO mice showed a progressive improvement in insulin sensitivity (0.5 to 2 yr) (**Figures 5D, E**).

### Aging CgA-KO mice maintain insulin signaling pathways

**Figure 6:**
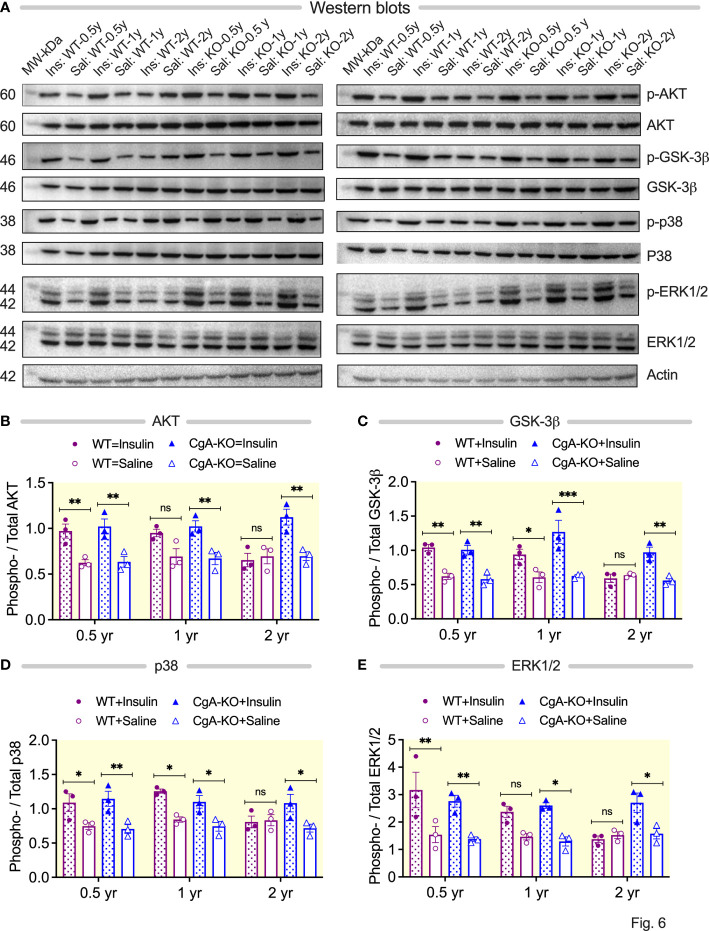
Aging CgA-KO mice maintain insulin signaling pathways. **(A)** Western blots of phosphorylated-AKT (S473), total AKT, phosphorylated GSK-3β (S9), total GSK-3β, phosphorylated p38 (T180/Y182), total p38, phosphorylated ERK1/2 (T202/Y204), and total ERK1/2 in the livers of 2 sets of aging WT and CgA-KO mice. **(B–E)** Densitometric analyses of the Western blots by 3-way ANOVA (n=3). **(B)** AKT (phosphorylated/total AKT). Age: ns; Genotype: p<0.01; Treatment: p < 0.001; Age x Genotype: p < 0.05; Age: x Treatment: ns; Genotype: x Treatment: p < 0.01; Age: x Genotype: x Treatment: p < 0.05. **(C)** GSK-3β (phosphorylated/total GSK-3β. 3-way ANOVA: Age: p < 0.01; Genotype: ns; Treatment: p < 0.001; Age: x; Genotype: ns; Age: x Treatment: p < 0.05; Genotype: x Treatment: p < 0.05; Age: x; Genotype: x; Treatment: ns. **(D)** p38 (phosphorylated/total p38). Age: ns; Genotype: ns; Treatment: p < 0.001; Age: x Genotype: ns; Age x Treatment: ns; Genotype: x Treatment: p < 0.05; Age x Genotype: x Treatment: p < 0.05. **(E)** ERK1/2 (phosphorylated/total ERK). Age: ns; Genotype: ns; Treatment: p < 0.001; Age x Genotype: ns; Age: x; Treatment: ns; Age x Genotype: x; Treatment: ns. *P < 0.05; **P < 0.01; ***P < 0.001.

Insulin promotes glucose uptake by signaling *via* the serine/threonine kinase AKT (52). While intraperitoneal injection with insulin did not result in increased phosphorylation of AKT (S473) in the liver of 2 yr old WT mice, it did so in 2 yr old CgA-KO mice (**Figures 6A, B**). Another important function of insulin is to promote glycogenesis through the activation of glycogen synthase kinase (GSK)-3β by AKT (52). Administration of insulin also did not result in phosphorylation of GSK-3β (S9) in the liver of WT mice, but it did in 2 yr old CgA-KO mice (**Figures 6A, C**). The mitogen-activated protein kinase (MAPK) pathway constitutes a second essential branch of insulin signaling, which is responsible for cell growth, differentiation, and survival (53). Insulin-stimulated phosphorylation of MAPK components p38 (T180/Y182) and ERK1/2 (T202/Y204) was compromised in the liver of aging WT mice (2 yr old), but not affected in aging CgA-KO mice (**Figures 6A, D, E**).

### Increased mitochondrial fusion in aging CgA-KO mice

**Figure 7:**
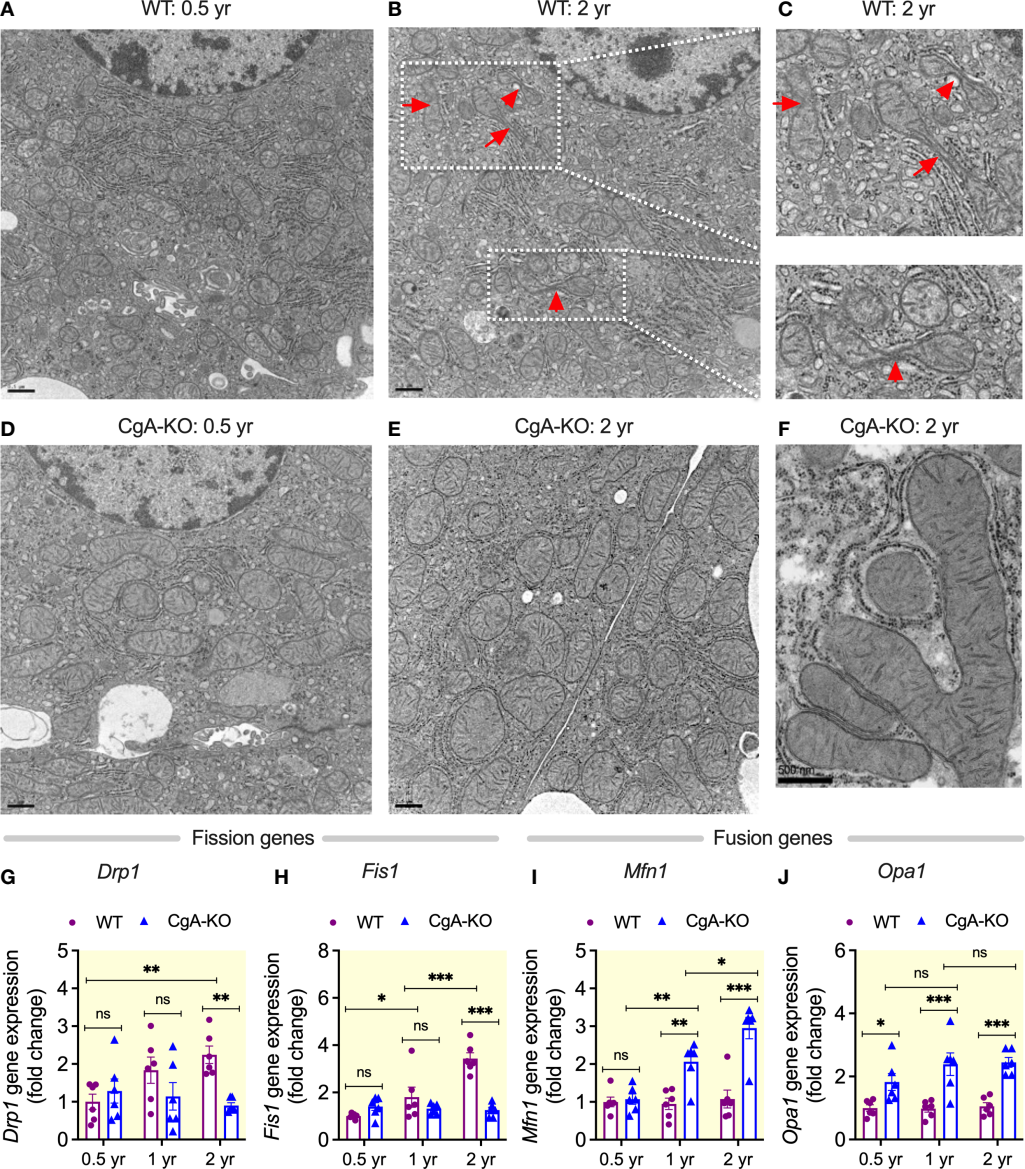
Increased mitochondrial fusion in aging CgA-KO mice. Electron micrographs of liver sections: **(A–C)** Mitochondrial fission in aging WT mice. **(D–F)** Mitochondrial fusion in aging CgA-KO mice. Scale bars: 0.5 µm **(A, B, D, E)**. Expression of mitochondrial fission genes in the liver of WT and CgA-KO mice: **(G)** *Drp1*. 2-way ANOVA: Interaction: p<0.05; Age: ns; Genotype: p<0.05. **(H)** *Fis1*. 2-way ANOVA: Interaction: p < 0.001; Age: p < 0.001; Genotype: p < 0.001. Expression of mitochondrial fusion genes: **(I)** *Mfn1*. 2-way ANOVA: Interaction: p < 0.001; Age: p < 0.001; Genotype: p < 0.001. **(J)** *Opa1*. 2-way ANOVA: Interaction: ns; Age: ns; Genotype: p < 0.001. *P < 0.05; **P < 0.01; ***P < 0.001.

Mitochondria frequently undergo coordinated cycles of fusion (elongation, more efficient respiration) and fission (shortening, less efficient), which are called mitochondrial dynamics, to meet specific cellular requirements (54), but these dynamics are compromised with age (55). Ultrastructural studies revealed increased mitochondrial fusion (by 1.9-fold) in liver cells of aging CgA-KO mice compared to increased fission (by 2.2-fold) in aging WT mice (**Figures 7A–F**). In line with this, expression of genes involved in mitochondrial fission *Drp1* (dynamin 1-like) and *Fis1* (fission, mitochondrial 1) were increased in the liver of 2 yr old WT mice, whereas the expression of genes involved in mitochondrial fusion *Mfn1* (mitofusin 1) and *Opa1* (mitochondrial dynamin like GTPase) were increased in aging CgA-KO mice (1 and 2 yr old) (**Figures 7G–J**). These experiments show that mitochondrial remodeling is oppositely affected in aging CgA-KO mice compared to WT mice.

### CgA-KO mice exhibit increased mitochondrial biogenesis

**Figure 8:**
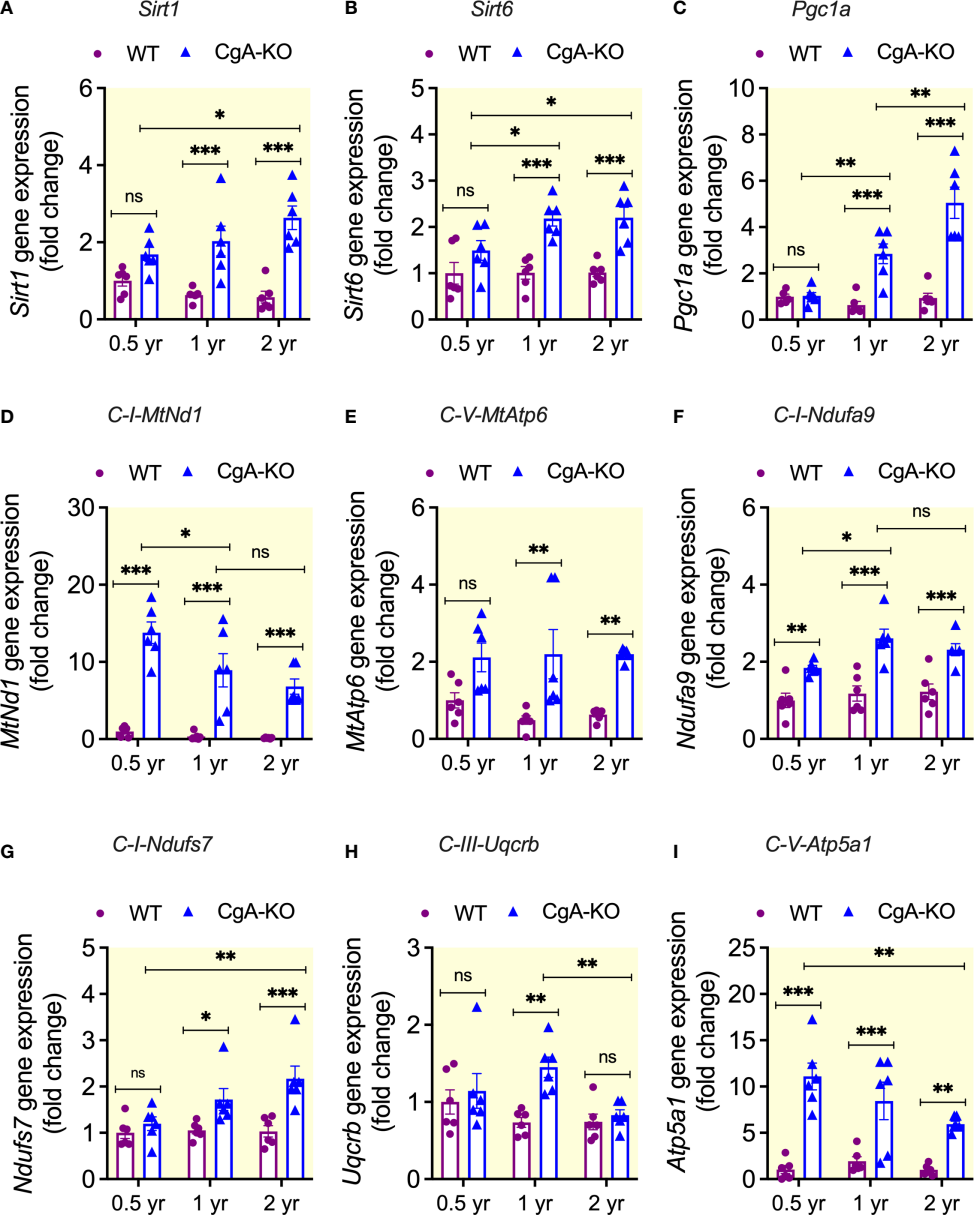
Increased expression of mitochondrial genes in CgA-KO mice. Expression of genes involved in mitochondrial biogenesis in the liver of WT and CgA-KO mice. 2-way ANOVA was used to analyze the following genes (n=6): **(A)** *Sirt1*. Interaction: p < 0.05; Genotype: p < 0.001; Age: ns. **(B)** *Sirt6*. Interaction: ns; Genotype: p < 0.001; Age: ns. **(C)** *Pgc1a*. Interaction: p < 0.001; Genotype: p < 0.001; Age: p < 0.001. Expression of mitochondrially encoded genes: **(D)** *MtNd1*. Interaction: p < 0.05; Genotype: p < 0.001; Age: p < 0.01. **(E)** *MtAtp6*. Interaction: ns; Genotype: p < 0.001; Age: ns. Expression of nuclear-encoded genes: **(F)** *Ndufa9*. Interaction: ns; Genotype: p < 0.001; Age: p < 0.05. **(G)** *Ndufs7*. Interaction: p < 0.05; Genotype: p < 0.001; Age: p < 0.05 **(H)** *Uqcrb*. Interaction: ns; Genotype: p < 0.01; Age: ns. **(I)** Atp5a1. Interaction: ns; Genotype: p < 0.001; Age: ns. *P < 0.05; **P < 0.01; ***P < 0.001.

Aging is associated with decreases in mitochondrial density, a result of decreased mitochondrial biogenesis (56). *Sirt1* (sirtuin 1) (57), *Sirt6* (sirtuin 6) (58), and *Pgc1*α (peroxisome proliferative activated receptor, gamma, coactivator 1 alpha) (59) are involved in mitochondrial biogenesis and their expression decreases with aging. Although the density of mitochondria was decreased (by 19.8%) in the liver of aging WT mice **Figures 7A, B**), there were no significant changes in the expression of these genes (**Figures 8A–C**). In contrast, aging CgA-KO mice showed an increased (by 12.6%) mitochondrial density (**Figures 7D, E**) and an increased expression of the *Sirt1*, *Sirt6*, and *Pgc1a* genes (**Figures 8A–C**).

### Differential expression of mitochondrial genes in WT and CgA-KO mice

Age-related mitochondrial deterioration (i.e., reduced mitochondrial DNA volume, integrity, and functionality) is associated with a decline in mitochondrial function (60). Therefore, we determined expression of mitochondria-encoded genes in aging WT and CgA-KO mice. While the expression of component of Complex I *MtNd1* (mitochondrially encoded NADH dehydrogenase 1) and Complex V *MtAtp6* (mitochondrially encoded ATP synthase 6) decreased in aging WT mice, the expression of those two genes remained consistently high in aging CgA-KO mice compared to WT mice (**Figures 8D, E**).

Nuclear-encoded proteins also play crucial roles in optimal functioning of mitochondria (61). Therefore, we also evaluated the expression of nuclear-encoded genes coding for mitochondrial components. The expression of Complex I *Ndufa9* (NADH: ubiquinone oxidoreductase subunit A9) and Complex I *Ndufs7* (NADH: ubiquinone oxidoreductase core subunit S7) were higher in aging CgA-KO mice compared to aging WT mice (**Figures 8F, G**). Increased expression of Complex III *Uqcrb* (ubiquinol-cytochrome c reductase binding protein) was evident in 1 yr old CgA-KO mice, but it declined in 2 yr old CgA-KO mice (**Figure 8H**). Finally, although CgA-KO displayed increased expression of Complex V *Atp5a1* (ATP synthase, H+ transporting, mitochondrial F1 complex, alpha subunit 1) from 0.5 yr to 2 yr compared to WT mice, there was a significant decrease in expression of this gene between 0.5 and 2 yr CgA-KO mice (**Figure 8I**).

### PST increases blood glucose and SBP in aging CgA-KO mice

**Figure 9:**
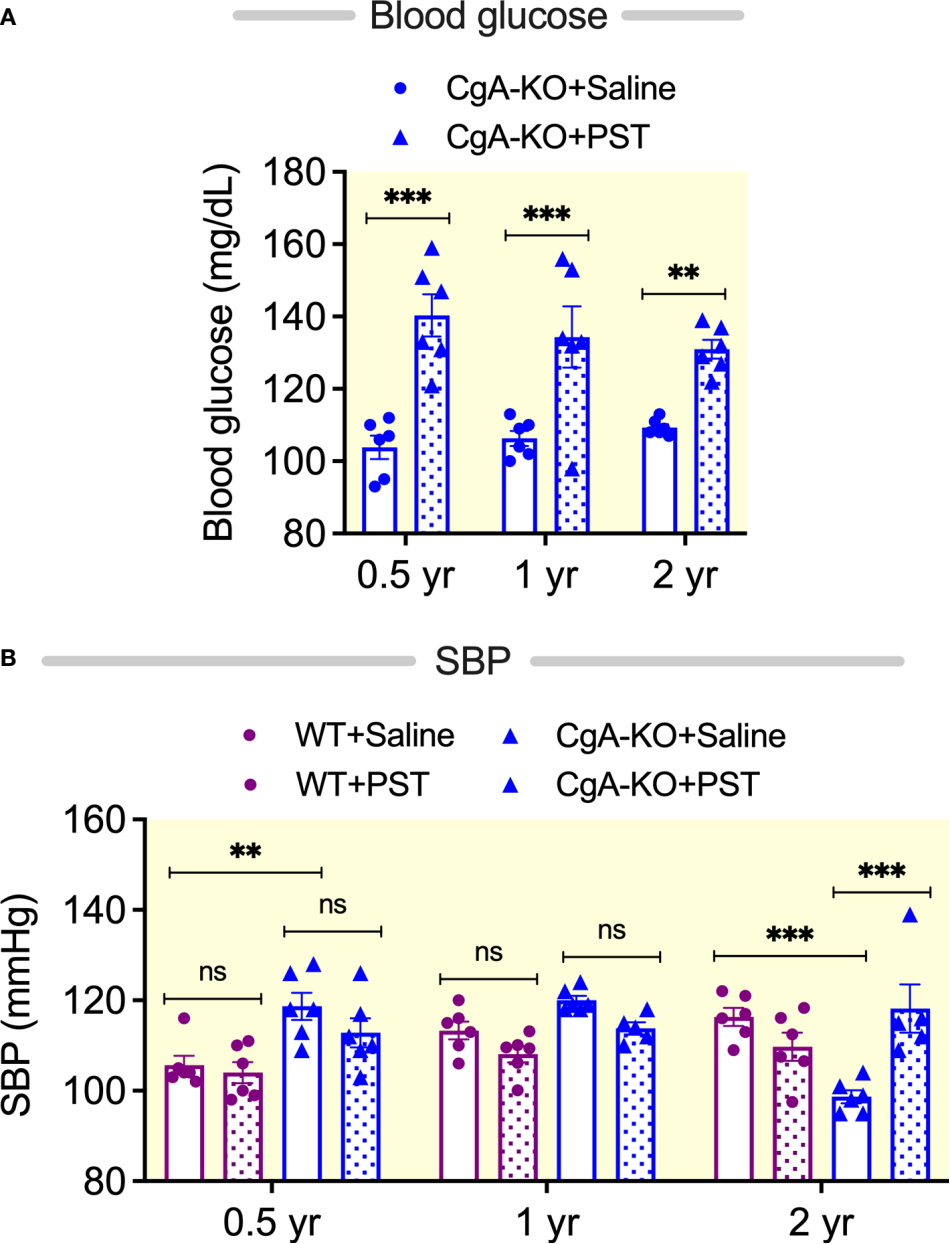
PST-induced changes in blood glucose and blood pressure. PST increases blood glucose in CgA-KO mice. **(A)** Blood glucose in PST (2 µg/g BW for 30 days intraperitoneal injection)-treated mice. 2-way ANOVA: Interaction: ns; Age: ns; Genotype & Treatment: p < 0.001. PST increases SBP in aging CgA-KO mice. **(B)** SBP in PST-treated mice. 3-way ANOVA: Age: ns; Genotype: p < 0.01; Treatment: ns; Age: x Genotype: p < 0.001; Age: x; Treatment: p < 0.01; Genotype: x; Treatment: p < 0.05; Age x; Genotype: x; Treatment: p < 0.001. **P < 0.01; ***P < 0.001.

We and others have previously reported that PST acts as an anti-diabetic peptide (14, 15, 62, 63). PST increases blood glucose levels in rodents by activating gluconeogenesis and glycogenolysis (14, 64). Since CgA-KO mice lack PST, we reasoned that supplementation of CgA-KO mice with PST would increase blood glucose levels. Indeed, intraperitoneal administration of PST (2 µg/g body weight/day for one month) increased blood glucose levels in 0.5, 1, and 2, yr old CgA-KO mice (**Figure 9A**). It has been recently shown that PST is associated with hypertension in humans (65). In congruence with these findings, we observed increased blood pressure after chronic supplementation of aging CgA-KO mice with PST (**Figure 9B**). Since PST is a pro-inflammatory peptide (15), the present findings also support inflammation-induced blood pressure in aging WT mice.

## Discussion

The precise mechanisms of aging are incompletely understood. Extensive research has led to the enumeration of hallmarks of aging including genomic instability, epigenetic alterations, loss of proteostasis, deregulated nutrient sensing, mitochondrial dysfunction, chronic inflammation and altered intercellular communication (66, 67). We focus our discussion on the role of CgA in inflammation, glucose metabolism, hypertension, and mitochondrial health, in the context of aging-related regulation of gut permeability on the spread of bacterial DNA and levels of Vsig4/CRIg.

### Bacterial DNA, Vsig4/CRIg and aging

The gut barrier separates trillions of microbes, microbial toxins, and food antigens from the lumen (outside of the gut) and the gut, the largest immune system in the body (68–70). A “leaky gut” allows entry of microbes and microbial products through the epithelium and causes systemic inflammation and associated diseases, including metabolic syndrome, neurodegenerative diseases, and cancer. Aging is associated with a compromised gut barrier (68–70). The widened tight junctions and adherens junctions in aging WT mice found in our study seem therefore consistent with the existing literature. We have recently reported that the high blood pressure in diet-induced obese (DIO) mice was due to the bacterial DNA-induced inflammation of the adrenal medulla and the consequent hypersecretion of norepinephrine (37). Other reports revealed a low-grade tissue inflammation owing to accumulation of bacterial DNA and the consequent development of insulin resistance (29, 71, 72). Therefore, accumulation of bacterial DNA in tissues apparently contributes to high blood pressure and decrease insulin sensitivity in aging WT mice.

The liver plays a critical role in filtering bacteria and their byproducts from the portal vein draining the intestine (29). The ability of the liver to remove bacterial components from the bloodstream is mainly attributed to phagocytosis by Kupffer cells (KCs), the resident macrophages of the liver (25, 26). KCs express Vsig4/CRIg, which is critical for KCs to efficiently bind and phagocytize complement C3-opsonized bacterial products (25, 27, 73). Therefore, the increased expression of Vsig4/CRIg in the liver of 2-year-old CgA-KO mice is most likely responsible for the efficient removal bacterial DNAs in the liver and the consequent decreased accumulation of bacterial DNA in most, if not all, organs. This is further aided by the tighter gut barrier in CgA-KO mice (74).

However, a limitation of this study is that not all results obtained in this study were from the same animals, and we were therefore unable to perform a correlation analysis.

### Effect of Vsig4/CRIg on inflammation, insulin resistance and cardiac function

One of the major and consistent changes that occurs during aging is the dysregulation of the immune response, leading to a chronic systemic inflammatory state (75). Even in the absence of overt inflammatory disease, healthy older people exhibit high serum concentrations of C-reactive protein (CRP) and other inflammatory mediators such as IL-6, IL-8, and TNF-α (76). This so-called inflammaging has been reported to be associated with an increased risk of mortality in healthy and frail older adults (76). It is not clear as to how a chronic inflammatory state develops with the progression of age. It is possible that the gut dysfunction due to enhanced permeability and low levels of Vsig4/CRIg are early events associated with aging. The resultant increased circulating and tissue bacterial DNA activates inflammatory pathways to remove the pathogens and pathogenic products that are not cleared in the liver. We found that many of the inflammatory phenotypes are reversed in CgA-KO, as we found decreased levels of TNF-α, IFN-γ and CCL2 in older CgA-KO mice. An increased expression of Vsig4/CRIg is known to suppress inflammatory pathways including reduced levels of TLR4, NLRP3 and IL-1β (77, 78). The elevated expression of *Vsig4* in aging CgA-KO mice might therefore explain “healthy aging” in CgA-KO mice.

We further show that GSIS and post receptor insulin signaling are compromised in aged mice. Inflammation and aging are long known to be closely associated with insulin resistance and glucose metabolism. Aging impairs insulin-mediated glucose uptake, and reduces the ability to suppress hepatic glucose output (49). It has been recently reported that the leakage of gut microbial DNA-containing extracellular vesicles (mEVs) results in a marked accumulation of bacterial DNA in metabolic tissues (e.g., liver, skeletal muscle, adipose tissue, adrenal medulla, and pancreatic islets) in obesity, eventuating in obesity-associated tissue inflammation and insulin resistance (29, 37, 72). Obesity suppresses Vsig4/CRIg+ expression in Kupffer cells in the liver, facilitating accumulation of bacterial DNAs in host tissues.

While post-receptor insulin signaling was compromised in WT mice, insulin signaling remained intact in CgA-KO mice. This is consistent with the report that Vsig4/CRIg activates AKT suppressing inflammation. The increased expression of Vsig4/CRIg in aging CgA-KO mice, therefore, likely not only resulted in more effective clearance of bacterial DNA, but also increased insulin sensitivity by activating the AKT and suppressing inflammatory pathways.

Age is a powerful risk factor for hypertension, and cardiovascular death (6, 7, 79–81). The present study shows that while aging caused hypertension in WT mice, high BP in 0.5-year-old CgA-KO mice was spontaneously reversed in 2-year-old CgA-KO mice. One of the factors that controls blood pressure is increased spillover of NE from sympathetic nerve endings in the heart and kidney. Increased spillover of NE in the heart (48, 82–85) and kidney (86–88) partly accounts for increased blood pressure in 2-year-old WT mice. Since inflammation-induced hypertension has been reported previously (13, 37), we believe that the bacterial DNA-induced inflammation of the heart may also contribute to the high blood pressure in aging WT mice. It therefore seems likely that the heart of aging CgA-KO mice shows less inflammation than the heart of aging WT mice, but his must be experimentally confirmed.

Conversely, spontaneous reversal of blood pressure in aging CgA-KO mice could be due to decreased spillover of NE in heart and kidney as well as increased expression of Vsig4/CRIg and the consequent removal of bacterial DNAs and diminution of inflammation. This is further substantiated by the increase in blood pressure in normotensive 2-year-old CgA-KO mice after treatment with the pro-inflammatory peptide PST.

### Mitochondrial dysfunction and senescence

Aging is associated with mitochondrial dysfunction, including decreased oxidative capacity with increased oxidative damage, and hyperglycemia induces mitochondrial fragmentation/fission with enhanced respiration and increased ROS production. Our data show that aging WT mice had increased mitochondrial fission, which may result in increased ROS production (89). In contrast, aging CgA-KO mice showed more mitochondrial fusion, a process by which damaged mitochondria may acquire undamaged genetic material and maintain functionality (90). Moreover, electron transport has been reported to be optimal for ATP production in elongated/fused mitochondria (91). Therefore, the mitochondrial organization and the increased mitochondrial biogenesis in aging CgA-KO mice likely support a healthy metabolism. In aging WT mice, the mitochondria are not only more fragmented, but their biogenesis is impaired owing to an age-associated decline in *Pgc1a* expression (92) and this is associated with a loss of mitochondrial content and function (92). It can therefore be expected that oxidative stress is higher and mitochondrial respiration is lower in aging WT mice compared to CgA-KO mice, although this need to be experimentally confirmed.

Other important proteins are the 13 mtDNA-encoded genes that are all components of the respiratory chain or the ATP synthase, and whose expression is directly related to oxidative phosphorylation (93). Notably, their expression reflected by complex I and IV activity decreases with age in the liver of mice (94). Consistent with this report, we found decreased expression of *MtNd1* and *MtAtp6* in aging WT mice, while the expression of those two genes remained consistently high in aging CgA-KO mice.

The mtDNA also has a higher rate of mutation (92) and has a higher rate of oxidative damage. There is a substantial (up to 40%) reduction in rat liver mitochondria of old animals (24 months) in comparison with young animals (3-4 months) (95). The current evidence suggests that mtDNA mutations and impaired OxPhos are primarily responsible for premature aging (96). Like the expression of the *MtNd1* gene, nuclear encoded complex I genes *Ndufa9* and *Ndufs7* are also overexpressed in aging CgA-KO mice compared to aging WT mice. Together, these findings suggest that the organization and function of mitochondria are improved in aging CgA-KO mice. Improved mitochondrial function in aging CgA-KO mice is possibly due to increased expression of Vsig4/CRIg as it has been recently shown that Vsig4/CRIg activates the PI3K-AKT-STAT3 signaling axis, which leads to PDK2 upregulation and activation, thus inhibiting mitochondrial pyruvate metabolism and suppressing mitochondrial reactive oxygen species secretion (97).

### CgA regulates healthy aging

In the current work, we show that CgA-KO mice represent a model for “healthy aging”. This data strengthens the notion that CgA is a target for treatment of aging-related diseases hypertension and metabolism. A key open question is whether the processing of CgA is altered upon aging. Proteolysis of CgA yields multiple peptides, including CST and PST which have opposite functions, at least in terms of glucose metabolism and regulation of blood pressure. Perhaps the conversion of CgA into these bioactive peptide fragments alters upon aging, for example due to altered expression of proteases. However, another possibility is that the processing does not alter but that downstream signaling alters upon aging. In addition, it will be important to translate these findings to human and determine whether CgA also plays a role in regulation of the gut permeability in human.

## Data availability statement

The data presented in the study are deposited in the NCBI Gene Expression Omnibus (GEO) repository, accession number GSE217145.

## Ethics statement

All studies with mice were approved by the UCSD and Veteran Affairs San Diego Institutional Animal Care and Use Committees and conform to relevant National Institutes of Health guidelines.

## Author contributions

ML, SS, SJ, KT, HG, ZJ, WM and SM researched data. ML, SS, WY, GG and SM analyzed the data. ML, GG and SM wrote the manuscript. SJ, WY, FM and GB participated in discussion and reviewed/edited the manuscript. SM conceived the study and made the graphics. ML, SS, SJ, KT, HG and SM are the guarantor of this work and take responsibility for the integrity of the data and the accuracy of the data analysis. All authors contributed to the article and approved the submitted version.

## Funding

This research was supported by a grant from the Department of Veterans Affairs (I01 BX003934 to SM) and the National Institutes of Health (R01 GM 085490 to GG; 1 R21 AG072487-01 and 1 R01 AI163327-01A1 to GG and SM; R00DK115998, R21HD107516, and R01DK125560 to WY). FM is a National Health and Medical Research Council Senior Research Fellow (GNT1060075).

## Acknowledgments

Transmission Electron Microscopy was conducted at the Cellular & Molecular Medicine Electron Microscopy Core Facility at UCSD.

## Conflict of interest

The authors declare that the research was conducted in the absence of any commercial or financial relationships that could be construed as a potential conflict of interest.

## Publisher’s note

All claims expressed in this article are solely those of the authors and do not necessarily represent those of their affiliated organizations, or those of the publisher, the editors and the reviewers. Any product that may be evaluated in this article, or claim that may be made by its manufacturer, is not guaranteed or endorsed by the publisher.
